# Progress in Medicine

**Published:** 1900-03-24

**Authors:** 


### Progress in Medicine.

### DISEASES OF THE NERVOUS SYSTEM.

(*Continued from page* 397.)

**Tumours of the Corpus Callosum.**—Schuffer[3] has collected 25 cases of tumours of the corpus callosum. Most of the cases occurred after 40 years of age, and the average duration of life was one year. Headache failed in 11 cases; vomiting was absent in 15; optic neuritis was observed in 10 cases, and absent in seven, not looked for in the others. The mental condition was always changed, generally in the direction of weakness of intellect and memory, somnolence, &c. Convulsions occurred in 11 cases. The mental changes appear to be much more constant in tumours of the corpus callosum than in tumours of other parts of the brain. When there are no definite localising symptoms in the presence of increasing failure of intelligence, one ought to think of a tumour in this situation.

**Loss of the Stereognostic Sense.**—Williamson[4] records a case of loss of the stereognostic sense, a condition in which there is no loss of sensation to tactile impressions, and yet the patient is unable to tell the nature of objects placed in the hand when the eyes are closed. A coin or other familiar object placed in the affected hand is felt, but (the eyes being closed) the patient fails to recognise what it is. Complete motor paralysis of the hand may be present, and yet the patient may be able to recognise the nature of many objects placed in contact with the affected hand; also when there is a considerable diminution, but not total loss, of tactile sensation in the hand, the stereognostic sense may be but little affected. This power of recognising objects held in the hand when the eyes are closed is probably an associative process dependent on tactile sensation, in the power of localising tactile impressions, and on muscular sense, and sometimes, in addition, on the sense of pressure, but it may be lost and yet the separate forms of sensation just mentioned be all intact. Though the exact seat of the lesion causing loss of the stereognostic sense has not been demonstrated pathologically, it is extremely probable that it is situated in the cortex of the parietal lobe just behind the arm centre of the ascending parietal convolution. The cases as yet recorded have been due to cerebral embolism and thrombosis, syphilis, and to injuries causing fractures of the parietal bone.

**Double Consciousness.**—Hyslop[5] brings forward observations which help to bridge the apparently impassable gulf between double consciousness and mere ordinary experiences. The cases fall under the following types: (1) Cases seen in childhood in which the condition is preceded by night terrors or by somnambulism, or by both. Thus a schoolboy aged 14 who had suffered from night terrors was found one night sharpening a knife with intent to kill; after a desperate resistance he was overpowered. Subsequently he remembered nothing whatever about the incident. (2) Types in which the abnormal states are preceded by profound sleep, and in which the normal state is reached after prolonged sleep. Thus a young lady on waking after a profound sleep was discovered to have lost every trait of acquired knowledge, and had to relearn everything. After a few months another fit of somnolency preceded her return to the normal state. She was as unconscious of her double character as each of two distinct persons is of the other's nature. (3) Types of alternating personality mainly dependent on alteration of memory, &c., due to traumatism and disease. After an injury or shock the power of recall of mnemonic images may be inhibited, and recent or remote events may, for the time being, be quite inhibited. Thus a woman aged 56 was knocked down by a cab, and afterwards fancied there were people in the room when there were really no people there. She also insisted she had been recently confined, and nursed the pillow as a baby. (4) Epileptiform types. Temporary loss of memory is often found after fits, may reach every grade, and may persist for several years. Thus, a female servant was morally defective and perverted in the abnormal after-stage, whilst in the normal state she had no memory of her vicious acts. (5) Insane types, where there is alternation of two personalities, the alternation may be complete or incomplete. (6) Anæsthetic hysterical types, in which there is a noticeable amount of anæsthesia, together with a temporary amnesia, which brings them within the category of double consciousness. (7) "Mediumship" types include those cases in which during the secondary state the subjects speak, write, or act as if animated by others, living or dead. The states vary from the occurrence of automatic writing to higher intellectual flights and theatrical scenes.

We may conclude from a consideration of these cases that, on the psychological side, in the abnormal states the permanent group or mass of feelings is only in part or slightly subject to the power of volition, and that there are wanting those conscious accompaniments —or processes of apperception—which raise the mental states into our self-consciousness. The condition is one of "sciousness" pure and simple. Apparently each unit of the highest evolved parts of the sensorium has its own separate element of consciousness, which exhibits itself under certain physical conditions as occurring within that unit. The amnesic defects which go far to constitute what we call double consciousness are due to failure of connections between neighbouring neurons which thus become isolated. It is possible that there are no end to the possible number of personalities in one individual.

**Paralysis Following General Anæsthesia.**—Leszynsky[6] is of opinion that paralysis following general anæsthesia is due to a lesion of the nerve trunks, and always the result of direct or indirect pressure on a nerve or nerves during the administration of an anæsthetic. The misleading terms "anæsthesia-paralysis" and "post-operative paralysis" should be dispensed with. The fact that in many alcoholic subjects the vulnerability of the peripheral nerves is sometimes increased to such a degree that a moderate amount of pressure may produce paralysis has led to the supposition that chloroform also produces toxæmia, whereby the peripheral nerves are rendered more susceptible to pressure. But the duration of the anæsthesia is short, and there is generally to be found on inquiry a history of long-continued pressure on the nerves affected during the operation. The paralysis, as a rule, affects the upper extremities, and is occasioned by any of the following conditions—prolonged elevation and extension of the arms, metal clamps or tight straps over the shoulders, extension of the head to one side, placing the arm under the head and retaining it there, or permitting the arm or leg to hang over the edge of the table. The importance of the condition is that, though it is usually quickly recovered from, it may in some cases persist for months or years.

**Adiposis Dolorosa.**—Hale White[7] records a case of this rare disease, the etiology of which is as yet obscure. So far all the observed cases have been in women, and generally between the ages of 40 and 60 years. The first thing noticed is that fatty masses appear on the trunk and limbs, often symmetrically. Fresh masses appear, and at the same time the patient becomes generally obese. The face, hands, and feet escape. Pain is a striking symptom. It is usually noticed as soon as, it may be, before the fatty masses; it may be felt in parts that do not become fat. It may be very severe, is often sharp burning or scalding, and frequently paroxysmal. The patient often complains early in the course of the disease of a sense of coldness, and if examined when the disease is fully established is usually found to have greatly diminished powers all over of appreciating pain, touch, heat, and cold. This diminution of sensation varies in different parts of the body in the same patient. Often the masses of fat are intensely tender when grasped. In some cases there has been great mental dulness; one patient died demented, and White's patient goes out of her mind temporarily. Headache is a common symptom. The mucous membranes, appetite, pulse, temperature, urine, speech, hair, and optic discs are normal. There is no œdema. In most cases the disease is progressive. One case improved on thyroid, in others it does no good. Dercum found that a piece of fat, removed during life, was of embryonic type, and one nerve fibre examined showed dense connective tissue, with a multiplication of nuclei. He suggests that the disease is a trophic neurosis. It may be that the disease is due to some alteration in the thyroid gland.

[3] Schuffer, Reviv. sper. di Freniatria, Vol. 27. [4] Williamson, Brit. Med. Jour., Dec. 9, 1899. [5] Hyslop, Brit. Med. Jour., Sept. 23, 1899. [6] Leszynsky, Med. Rec., Oct. 21, 1899. [7] Hale White, Brit. Med. Jour., Dec. 2, 1899.

### DISEASES OF CHILDREN.

**Juvenile General Paralysis.**—On this subject Dr. Mott[1] has published notes of 22 cases, with 16 post-mortem examinations, and his researches, taken together with those of Thiry and Alzheimer on the Continent, seem to have established the identity of this disease with that occurring in later life. While Dr. Mott admits that there may be many exciting causes, he finds that hereditary syphilis is nearly always the predisposing cause. As regards the mental symptoms, he points out that many of the patients were mentally deficient from birth, while others were healthy up to a certain point, when symptoms of mental enfeeblement gradually developed. In all there was progressive dementia, accompanied in some by acute mania, or depression, or delusions of persecution or of grandeur, the last-mentioned not being nearly so common as in the adult form. As regards the motor symptoms, progressive paresis was noted in every case except one, and the other leading changes were affections of written and verbal speech, pupil phenomena, and altered knee jerks. Viewing the cases clinically, he divides them into two classes or types. In the first a mentally deficient child or a congenital imbecile suffering from fits at some period, varying from the age of eight years onwards, develops new symptoms of mental trouble, which become progressively worse, and are associated with progressive motor failure. The other type is when a normal child, even a bright, intelligent child, about the age of puberty or afterwards, becomes affected mentally, and shows a gradual progressive dementia and paresis. In the 16 post-mortem examinations the characteristic lesions of general paralysis were found. The fact that obvious syphilitic arteritis is not present supports the view that the disease is parasyphilitic or metasyphilitic, there being a premature decay or degeneration of the nervous structures.

**Achondroplasia, Cretinism, and Mongolian Imbecility.**—These three affections present certain similarities which may lead to a mistaken diagnosis. Achondroplasia or chondro-dystrophia (Kaufman) is a disease affecting especially the long bones, and has been erroneously described by some writers as "congenital" or "fœtal rickets." Mr. W. Turner[2] gives the leading characteristics as follows: The growth of the child is stunted, the shortening being mainly due to the condition of the legs. The articular portions of the limbs are irregular in size and shape, but the diaphyses are extremely short. The arms are similarly short, so that even when hanging down the fingers can scarcely reach the iliac crests. The fingers are tapering, and the index and middle fingers are widely separated from the ring and little fingers. The face is small, the nose is depressed, and the calvarium is relatively very large. A well-marked lordosis is present in the lumbar region. The defects in structure are due to changes in the bones which grow in cartilage, and are probably begun and ended in fœtal life between the third and the sixth month. Although the general appearance may suggest cretinism, there is an absence of any mental affection, of supraclavicular pads, of the coarse skin and hair, of the large protruding tongue, and of any improvement under thyroid medication. Certain similarities between these two affections and the type of imbecility known as Mongolian may lead to further confusion. Patients of the last class, according to Dr. G. A. Sutherland,[3] have special characteristics, mental and physical, by which they may be distinguished. The skull is brachycephalic, with sunken bridge of the nose, and orbital fissure obliquely formed, so that the palpebral fissure, which is small, slopes inwards and downwards. The peculiar aspect of the face produced by these changes suggested the term "Mongolian." The tongue is large, and is often protruded. The little finger is abnormally short, and is curved outwards. The large and small joints are loosely strung, and allow of a much wider range of movement than usual. Congenital cardiac disease is frequently present. The mental characteristics are those of much-delayed development, talking, walking, and co-ordinating power being learned at a comparatively late age. Discussing the pathology of this affection he suggests that there is probably some cerebral defect dating from an early period of fœtal life, and, from the large number of cases in which hereditary syphilis has been present, he thinks that parental syphilis may be the cause. This affection is to be distinguished from cretinism by its congenital nature, by the active movements of the infant, as contrasted with the dull, impassive state of a cretin, by the changes in the skull producing the characteristic facial aspect, and by the absence of any benefit from thyroid treatment. Both these papers are fully illustrated with typical examples of the diseases referred to.

**Cerebral Diplegia.**—Dr. James S. Collier[4] has recorded some cases of this affection, and given a good summary of the present knowledge of the subject. The pathology is still obscure, probably because different factors are at work in different cases. The initial morbid processes are stated to be—(1) degeneration of the neurons of the cerebral cortex; (2) vascular lesions, embolism, thrombosis, and intra-cerebral hæmorrhage; and (3) perhaps meningeal hæmorrhage. These initial lesions are seldom seen because the result is not fatal, and it is not perhaps for years that an opportunity presents itself for determining the exact condition of the brain. The lesions then found are—(1) atrophy of the convolutions; (2) porencephaly; and (3) no coarse lesion, but degeneration and disappearance of the cortical neurons. The common form of bilateral spastic hemiplegia is connected with deep lesions of the hemispheres, while the other forms of diplegia, such as the athetotic and choreic, are connected with superficial lesions of the hemispheres. Although a cure cannot be expected, much improvement will follow from careful mental training, the use of appropriate gymnastic exercises, and, if necessary, the division of tendons.

**Peripheral Neuritis Following Chorea Treated with Arsenic.**—A report on this subject by Dr. Railton,[5] based on his experiences at the Manchester Clinical Hospital, is well worthy of perusal. Four cases in which the above sequence of events took place are related. The total amount of arsenic taken corresponded in the respective cases aged 12 years, 11 years, 10 years, and 10 years, to 6·3 grains arsenious acid, 8·4 grains, 8·1 grains, and 8·1 grains, the periods of administration being three weeks, four weeks, three weeks, and 24 days. Of these four cases, three suffered severely and one very slightly, but all eventually made a good recovery. The total number of cases of chorea treated in the hospital during the past 10 years was 312, in most of whom arsenic had been the remedy employed; and while in some patients it had to be stopped because of vomiting, &c., the above four represent the only examples of neuritis following its use. This result may supervene without any indication during the administration of the drug, and from a week to a fortnight subsequent to its discontinuance. As the result of his experience, Dr. Railton urges that no aggregate dose amounting to more than four grains of arsenious acid be administered to a child during an attack of chorea.

[1] Archiv. Pathol. Labor., Lond. County Council, 1899. [2] Practitioner, Sept., 1899. [3] Ibid, Dec., 1899; and the Lancet, Jan. 6, 1900. [4] Brain, Part lxxxvii., 1899. [5] Med. Chron., 1900, vol. ii., No. 5.

